# 3D Printing, Histological, and Radiological Analysis of Nanosilicate-Polysaccharide Composite Hydrogel as a Tissue-Equivalent Material for Complex Biological Bone Phantom

**DOI:** 10.3390/gels9070547

**Published:** 2023-07-05

**Authors:** Petar Valchanov, Nikolay Dukov, Stoyan Pavlov, Andreas Kontny, Tsanka Dikova

**Affiliations:** 1 Depatment of Anatomy and Cell Biology, Medical University of Varna, 9002 Varna, Bulgaria; stoyan.pavlov@mu-varna.bg (S.P.); andreas.kontny@mu-varna.bg (A.K.); 2 Department of Medical Equipment, Electronic and Information Technologies in Healthcare, Faculty of Public Health, Medical University of Varna, 9002 Varna, Bulgaria; ntdukov@mu-varna.bg; 3 Department of Dental Material Science and Prosthetic Dental Medicine, Medical University of Varna, 9002 Varna, Bulgaria; tsanka.dikova@mu-varna.bg

**Keywords:** hydrogels, 3D printing, scaffolds, phantoms, histological analysis, radiological analysis, nanosilicates, polysaccharides

## Abstract

Nanosilicate-polysaccharide composite hydrogels are a well-studied class of materials in regenerative medicine that combine good 3D printability, staining, and biological properties, making them an excellent candidate material for complex bone scaffolds. The aim of this study was to develop a hydrogel suitable for 3D printing that has biological and radiological properties similar to those of the natural bone and to develop protocols for their histological and radiological analysis. We synthesized a hydrogel based on alginate, methylcellulose, and laponite, then 3D printed it into a series of complex bioscaffolds. The scaffolds were scanned with CT and CBCT scanners and exported as DICOM datasets, then cut into histological slides and stained using standard histological protocols. From the DICOM datasets, the average value of the voxels in Hounsfield Units (HU) was calculated and compared with natural trabecular bone. In the histological sections, we tested the effect of standard histological stains on the hydrogel matrix in the context of future cytological and histological analysis. The results confirmed that an alginate/methylcellulose/laponite-based composite hydrogel can be used for 3D printing of complex high fidelity three-dimensional scaffolds. This opens an avenue for the development of dynamic biological physical phantoms for bone tissue engineering and the development of new CT-based imaging algorithms for the needs of radiology and radiation therapy.

## 1. Introduction

Bones are fascinating structures in the human body, providing support as well as structure, mobility, and protection. Understanding the intricate properties of bone tissue is crucial for advancements in fields such as pathology, histology, and radiology. In recent years, the revolutionary technology of 3D printing has emerged, allowing us to create realistic models of bones known as anthropomorphic phantoms. These phantoms mimic the spatial, physical, and biological characteristics of bone tissue, enabling us to study and develop innovative techniques in various scientific disciplines.

- Each type of phantom requires specific materials that closely simulate the desired properties of bone tissue. Spatial and biomechanical properties are represented by high-fidelity anatomical models [1] or benchmark devices for biomechanical testing [2,3] that require materials with minimal thermal deformation (thermo- or photopolymers), and can be 3D printed with FDM (fused deposition modeling), SLS (selective laser sintering), or SLA (stereolithography) at optimal spatial resolutions.
- Biological and optical properties are represented by bioscaffolds [4] or organ-on-chip devices [5] made of biomaterials such as hydrogel biopolymers with optimal cell culturing characteristics and staining properties that do not impede histological examination and analysis; these can be 3D printed with extrusion-based 3D printing.
- Radiological properties are represented by imaging phantoms [6], which require materials with an atomic mass and X-ray attenuation coefficient similar to that of natural bone (for instance, thermopolymers or polymer-inorganic clay composites); these can be 3D printed with FDM.

The aim of the present study is to develop a 3D printed composite hydrogel with optimal osteogenic and osteoconductive properties, staining qualities, and radiological properties that are similar to those of trabecular bone. By achieving this, we can fabricate complex biological phantoms that accurately represent physiological and pathological processes in bone tissue. Such devices allow the development of specific X-ray, CT (computed tomography), and CBCT (cone beam computed tomography) imaging algorithms with improved diagnostic value and reduced false negative results for a broad spectrum of bone-related pathological conditions.

### 1.1. Composite Polysaccharide–Nanosilicate Hydrogels

Hydrogels are a class of hydrophilic materials composed of polymers and water [7]. These polymers can form insoluble fiber networks mimicking the structure of the extracellular matrix of certain human tissues [8]. Polymerization of monomeric units is achieved by means of a crosslinking mechanism based on thermal, light, or ionic interactions [9]. Organic and inorganic additives (such as metallic or clay nanoparticles [10,11], carbon nanomaterials [12], growth factors [13], pharmacological substances [14], etc.) can substantially modify the initial properties of the pure material [15]. Certain combinations of polymers and additives are especially promising for the cultivation of specific mesenchymal tissues, including bone [16], cartilage [17], ligaments [18], muscles [19], blood vessels [20], and more. Laponite is a nanosilicate clay composed of 10 nm crystals with a discoid shape [21]. When dissolved in water, the two sides of the discoids acquire opposite polarities and spontaneously assemble into a “house of cards” configuration [22]. When added to a polysaccharide hydrogel, Laponite significantly improves both its 3D printability and its osteogenic properties [23]. These properties make nanosilicate composite hydrogels a promising tissue-equivalent material for the generation of complex three-dimensional structures with high spatial, biological, and radiological fidelity.

### 1.2. 3D Printing of Bioscaffolds

Composite hydrogels can be 3D printed into complex and highly porous three-dimensional bioscaffolds [4]. They are currently being developed from highly biocompatible polymers such as alginate [24], cellulose [25], silk, chitosan [26], hyaluronic acid [27], collagen [28], fibrin [29], etc., and modified with additives [12] to further improve their biological properties. The bioscaffold can be inoculated with cells and cultivated in a bioreactor with cell culture medium to establish a 3D cell culture with specific spatial characteristics [30,31]. The materials of the scaffold, the cell culturing media, the conditions in the bioreactor, and the cell type all determine the fate of the cells, and can be used to simulate a broad spectrum of physiological or pathological conditions in a biological phantom. Bioscaffolds can be used to generate new implantable synthetic tissues [32] and simulate rare pathological conditions [33], as well as in oncological diseases [34] and drug testing [35].

The main goal of bioengineering is the creation of synthetic tissue and organ transplants that can replace damaged organs and tissues [36]. This achievement will eliminate the shortage of organs for transplantation, which is the main issue in regenerative medicine. There are several factors that determine the ideal implantable bioscaffold [37]:

- Biocompatibility: the scaffold must provide the necessary base for adequate cellular adhesion, proliferation, and differentiation [38]. If the scaffold is implantable, it should not cause any inflammatory or immune reaction, which disrupts tissue regeneration and may cause rejection by the recipient.
- Bioresorption: the materials of the scaffold must be bioresorbable and eventually replaced by a newly generated extracellular matrix [39]. The byproducts of biodegradation should be nontoxic and easy to eliminate from the organism without interference with other organs and systems.
- Mechanical properties: the scaffold should possess mechanical properties corresponding to those of the tissue in which it will be implanted [40] and must preserve its integrity from the moment of implantation to the completion of the remodeling process. This condition is especially important for bone and cartilage engineering.
- Scaffold architecture: the scaffold should possess a porous structure specific to the engineered tissue, with interconnected spaces occupying a sufficient part of the total volume [41]. High porosity ensures adequate cell migration, diffusion of nutrients, and elimination of waste products. Adequate vascularization of the scaffold prevents necrosis, inflammation, and rejection of the implant [42]. Another key concern is cell adhesion, as cells bind to chemical groups (ligands) that are naturally present only in extracellular fibrillar glycoproteins. In non-natural materials, active adhesion sites can be engineered by adding binding sequences (such as Arg-Gly-Asp, RGD) or by other means to facilitate cell adhesion [43].
- Radiological properties: as an implantable structure, the bioscaffold should be controlled using imaging methods. This requires tissue-equivalent radiological properties that ensure proper control over scaffold implantation [44].
- Histological properties: staining qualities must ensure that the engineered matrix does not interfere with histological and cytological analysis during scaffold development and testing [45].
- Manufacturing technology: bioscaffold production with 3D printing or other spatially controlled technology requires high reproducibility as well as proper quality control and certification [46].

Composite nanosilicate hydrogels possess properties that fit well with the listed paradigm and that can be manufactured into reproducible complex porous bone-mimicking bioscaffolds through extrusion-based 3D printing. This makes them a promising material for bone tissue engineering.

### 1.3. 3D Printing of Imaging Phantoms

Medical imaging using CT, MRI, or ultrasound plays a vital role in diagnostics and research. To ensure accurate and high-quality images while maintaining patient safety, medical imaging phantoms have traditionally been employed. Thanks to their known material composition and simple geometries, such phantoms have become essential for quality assurance and standardization. However, traditional phantoms have limitations such as restricted material usage and limited geometric complexity. While more sophisticated alternatives exist, their high cost makes them less accessible.

The rapid advancement of 3D printing technology in recent years has led to a significant breakthrough in the domain of medical imaging phantoms [47,48,49]. The 3D printing of sophisticated geometries using diverse materials can facilitate the development of affordable anthropomorphic phantoms. These phantoms are crafted using tissue-equivalent materials to realistically and accurately depict organs specific to each imaging modality employed. Simple shapes are no longer a limitation, as intricate anatomical structures can be printed with diverse materials.

Anthropomorphic phantoms can be used for standard quality assurance procedures as well as for protocol optimization tasks, image reconstruction algorithm optimization, and testing of new emerging techniques.

One of the most accessible and popular 3D printing technologies is fused filament fabrication (FFF), more commonly known by its trademark name of fused deposition modeling (FDM). The specifics of the process mean that FFF printers can easily be modified and augmented for printing with various materials. Such is the case with bioprinting, in which the typical extrusion system is replaced by a motor-driven syringe [50]. These improvements have led to the next big challenge in modern biophysics and radiology, namely, the development of universal and multimodal anthropomorphic phantoms.

### 1.4. Development of Complex Multipurpose Biological–Radiological Phantoms

The different phantoms represent only specific properties of the target tissue: either biological, simulated by bioscaffolds and 3D cell cultures, or radiological, simulated by imaging phantoms. Certain advanced phantoms can represent several submodalities of the main property; for example, there are complex X-ray/CT/CBCT/Angio-CT phantoms. Recently, the possibility of a new kind of phantom has emerged: a complex cell-laden bioscaffold with a porous structure that represents the morphological, physiological, histological, and radiological properties of bone tissue. This type of device can simulate a wide range of physiological and pathological conditions, including osseous callus formation, bone remodeling, osteoporosis, osteosclerosis, bone cysts, osteodegenerative conditions, primary or metastatic bone tumors, etc. Their high radiological fidelity could make possible the development of specific imaging algorithms for the detection and differential radiological diagnosis of these conditions.

## 2. Results and Discussion

The goal of the present study is the development of a bioink for extrusion-based 3D printing of complex porous bioscaffolds with improved 3D printability, osteogenic, and radiological properties, and of the corresponding protocols for cytological, histological, and radiological analysis. The studied composite polysaccharide–nanosilicate hydrogel uses alginate and methylcellulose as a base and laponite as an additive. The heteropolysaccharides provide a three-dimensional interconnecting network of hydrophilic fibers, while the “house-of-cards” nanostructure of the laponite [22] improves stability during extrusion and increases osteoconductivity and osteogenic properties.

### 2.1. Preparation of the Hydrogel

For our experiments, we used a hydrogel containing alginate, methylcellulose, and laponite according to the methodology of Ahlfeld et al. [51] During the development of the optimal bioink recipe, we tested the main formula (Modification 1, M1) and a modification with an increased concentration of the ingredients (Modification 2, M2) (Table 1).

Modification 1 was the first recipe that we tested; however, our results of the 3D printing tests were unsatisfactory, as our 3D printer was unable to create a stable object which satisfying the aim of our study, namely, a bioink suitable for generating complex three-dimensional scaffolds. As it is well established that increased laponite concentration facilitates the formation of a self-supporting nanostructure in the composite [52], in Modification 2 we increased the concentrations of all the ingredients. The resulting hydrogel demonstrated excellent stability during extrusion, and was used successfully for the 3D printing of complex three-dimensional scaffolds.

The main issue during the preparation of the bioink was the formation of insoluble precipitates in the hydrogel. To achieve optimal conditions for solution of the compounds, we added absolute ethanol to the mixed powdered ingredients drop by drop until a slurry was formed. The mixture was added then to distilled water at 60 °C and stirred with a homogenizer for 10 min. The main ingredients are insoluble in ethanol and do not swell, which permits the formation of a homogeneous mixture. However, ethanol is highly hygroscopic and facilitates rapid penetration of water into the mixture during the solution phase. The resulting hydrogel was clear and thixotropic, with medium viscosity. After its preparation, the hydrogel was loaded into 10 mL syringes and stored for 24 h at a temperature of 4 °C. Any formed air bubbles were removed by centrifuging the syringes at 3000 rpm for 1 min.

We crosslinked the hydrogel with a 0.1 mol/L CaCl_2_ solution, which accelerates ionic coordination of alginate. For cross-link testing, samples of modifications M1 and M2 were prepared and extruded into quadrangular molds. The labeled molds with hydrogel were submerged in CaCl_2_ solution for 20 min and incubated in a thermostat at 36 °C for 20 min. After cross-linking, the M1 and M2 molds were stored at 4 °C for 24 h. The hydrogel blocks were removed from the molds and their crosslinking was assessed.

Modification 1 crosslinking was insufficient, and was improved slightly by the addition of Na_2_SO_4_, which changes the optimal crosslinking temperature of methylcellulose from 60° C to 36° C. Nonetheless, the test objects did not keep their spatial characteristics after crosslinking. Together with the poor results of the 3D printing tests, this was one of the main reasons for creating and testing a new modification with increased concentration of the ingredients.

The crosslinking of Modification 2 was excellent even without the addition of Na_2_SO_4_. After 10 min submersion into a 0.1 mol/L CaCl2 solution, the 3D printed objects solidified into a stable and resilient object without deformation. The structural stability of the crosslinked objects was the main reason for declaring the results of the crosslinking tests “excellent”. 

### 2.2. 3D Printing of Test Models and Complex Scaffolds

First, the hydrogel samples were loaded into the modular head of the 3D printer and the material was extruded to ensure the proper flow of the hydrogel through the nozzle. M1 oozed through the nozzle because of its low viscosity, and caused significant soiling of the building platform in the following 3D printing tests.

**Test 1. Simple geometric figures: calibration squares, cube, and cylinder.** For the first tests, several 3D models were prepared using Autodesk Meshmixer [53]:

- Square prisms of 40 × 40 × 10 mm, 30 × 30 × 10 mm, and 20 × 20 × 10 mm;
- A cube with a size of 20 × 20 × 20 mm^3^;
- A cylinder with a diameter of 20 mm and height of 20 mm.

**Figure 1:**
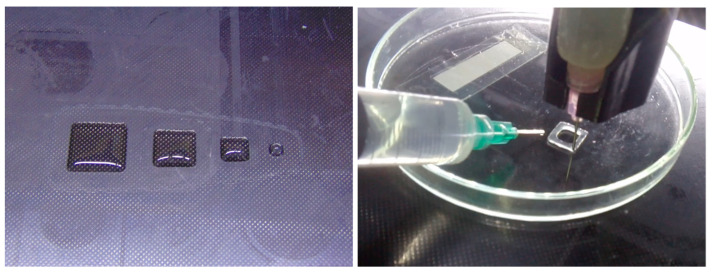
3D Printing Test 1—simple geometric figures: squares (**left**) and cylinder (**right**). These objects were 3D printed with Modification 1.

The models were exported as an stl file and sliced with Slic3r. The g-code was executed with Hyrel 3D Repetrel. The test objects were 3D printed with Modification 1 in a petri dish. After completion of the printing process, the objects were crosslinked with CaCl_2_ solution for 10 min at ambient temperature and washed with distilled water (Figure 1).

The M1 square prisms were unstable and unable to keep their shape for more than a few minutes. After dousing with CaCl_2_ solution, the objects deformed into an amorphous mass.

The first layers of the cube and the cylinder were printed with excellent quality, but during the second layer the objects started to lose shape, even after dousing with CaCl_2_ solution. During the third layer the objects became an amorphous mass, and the experiment was terminated.

After these unsatisfying results, Modification 1 was declared unsuccessful, and the following tests were performed with Modification 2.

**Test 2: Quadrangular scaffold.** A 3D model of a complex scaffold was prepared with Autodesk Fusion 360 [54]. The model consisted of interconnecting perimeters in a quadrangular grid with a length of 2 mm and height of 2 mm, forming 2 × 2 mm openings. The overall dimensions were 42 × 30 × 8 mm. A second version of the same model was generated with a perimeter height of 1 mm and overall dimensions of 42 × 30 × 4 mm. Both models were exported as stl files and 3D printed with preparation M2. A final version of the model was printed with the same parameters. The 3D printed objects were labeled Scaffold 1, Scaffold 2, and Scaffold 4.

**Figure 2:**
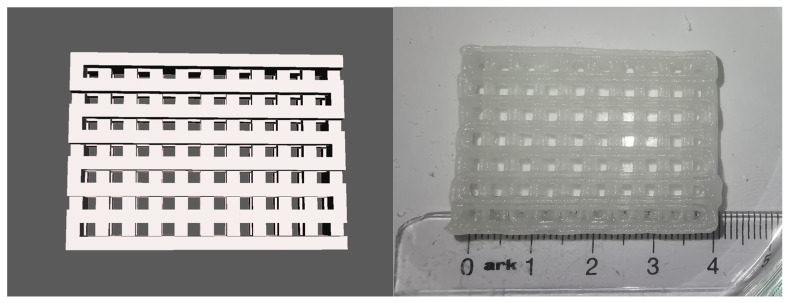
3D Printing Test 2—Quadrangular scaffold: 3D model (**left**) and 3D printed object (**right**). The objects were 3D printed with Modification 2.

The model was 3D printed with high dimensional accuracy, without deformation of the interconnecting parts and with proper overhangs and bridges. After 3D printing, the object was cross-linked using a solution of CaCl_2_ for 10 min. The model kept its dimensional accuracy and crosslinked into a solid object without deformation (Figure 2). The finished object was stored at 4 °C in a wet chamber for four days, and showed no dimensional deformation or hydrogel degradation.

**Test 3: Haversian system.** For the final test, a 3D model of a Haversian system (osteon) was prepared with Autodesk Fusion360. The model consisted of a central canal with a diameter of 1.5 mm representing the Haversian canal, and five concentric lamellae with a width of 1.5 mm and an offset between them of 1.5 mm. Eight radial channels with a diameter of 1.5 mm, representing the Volkmann channels, connected the central canal and the periphery of the model. The resulting structure was cylindrical, with a diameter of 28.5 mm and height of 10 mm, and was 3D printed with hydrogel M2 to confirm its 3D printability. The 3D printed object was called Scaffold 3.

**Figure 3:**
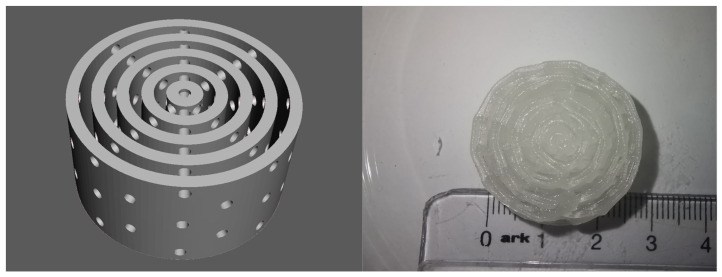
3D Printing Test3—Haversian System: 3D model (**left**) and 3D printed object (**right**). The objects were 3D printed with Modification 2.

The model was 3D printed with high dimensional accuracy; the overhangs and bridges were printed properly, and the channels and the offsets between the laminae were passable for liquids. After crosslinking with CaCl_2_ solution for 10 min, the 3D printed object solidified into a stable structure without deformation (Figure 3). This 3D printing test confirmed the results of Test 4, and Modification 2 was declared successful. Further experiments (CT scans and histological slides) were performed on the scaffolds 3D printed with Modification 2.

The overall results of the 3D printing tests demonstrate the importance of the polymer concentration in the hydrogel composite for extrusion-based 3D printing. A higher concentration of the carbohydrate polymers provided enough stability to ensure 3D printing with high dimensional accuracy (Table 2). At the same time, the increased laponite concentration allowed for the formation of a supporting nanostructure [52] inside the gel, significantly improving the quality of the 3D printing and the crosslinking of the hydrogel. Without an additional supporting medium (e.g., FRESH printing [55]), the lower concentrations of the ingredients were not sufficient to ensure the proper mechanical stability and dimensional accuracy of the finished objects.

### 2.3. Staining and Histological Properties of the Hydrogel

A histological analysis of the staining properties of Modification 1 with Hemalaun and Eosin (H&E) was described in a preceding article [15]. In the present study, we tested the effects of three common staining protocols on Modification 2.

**Figure 4:**
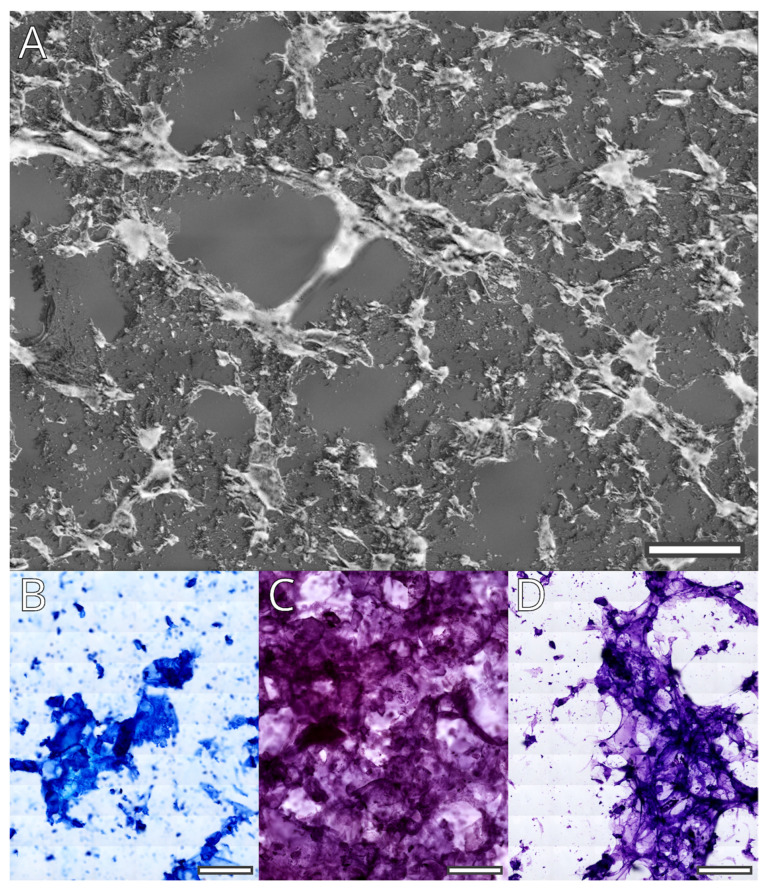
Microscopic examination of the cured hydrogel matrix. Fused mosaic images of dark field observation of the unstained hydrogel (**A**) and bright field images of the stained matrix: (**B**) Diff Quik stain; (**C**) Hemalaun–Eosin; and (**D**) Cresyl violet. All images were processed to reduce stitching artifacts and brightness, and contrasts were shifted to improve visual quality. The length of each scale bar corresponds to 250 μm.

Fifteen slices, each of one micrometer thick, were cut from the cured scaffolds manufactured with Modification 2 hydrogel. The unstained gel structure was observed with phase contrast and dark-field microscopy (Figure 4A). The gel formed a porous mesh surrounding interconnected spaces with diameters in the range of less than 100 μm and up to 500 μm. 

The three chosen staining protocols (Diff Quik, Cresyl violet, and Hemalaun–Eosin) are widely used for cytological and histological analysis. During the processing and passage through the liquid reactives, the gel is partially extracted. What remains is a flaky substance that is stained with variable strength by the basic dyes in the protocols (Figure 4B–D). This is probably the result of laponite acting as a mordant. In addition to the laponite containing flakes, Cresyl violet stains part of the clear remaining gel. Cresyl violet seems to stain the gel that does not contain laponite flakes as well; this, however, seems to be repaired by increasing the time for differentiation of the stain. 

The staining of the gel remnants creates a problematic environment for histological and cytological analyses of the inoculated bioscaffolds. However, as the gel is easily extracted during rehydration, longer washing in this phase of staining might be sufficient to reduce its influence. Additionally, our previous tests with Modification 1 suggest that preprocessing with citrate or chelating agents may reduce background staining by Hemalaun, and probably other nuclear dyes as well. Finally, the flaky appearance of the remaining laponite-bound gel is morphologically different from the cellular structure; thus, it can be easily recognized by an expert. Additionally, the unstained hydrogel is autofluorescent over a wide range of excitation wavelengths (image not shown). This imposes a significant problem, as it may impede detailed analysis of the phenotype of cultured cells by immunofluorescence. This issue requires further analysis.

### 2.4. CT Scanning and Radiological Analysis of 3D-Printed Hydrogel Scaffolds

Four objects 3D printed during the previous experiment were selected for radiological analysis: Scaffold #1, Scaffold #2, Scaffold #3, and Scaffold #4. They were scanned with a clinical CT scanner and a dental CBCT scanner. The resulting datasets were exported in DICOM format and their minimum, maximum, and mean values were calculated, along with the standard deviations in Hounsfield Units (HUs). The results were compared to those of a clinical CT scan of a dry cadaveric human calcaneus. 

**Figure 5:**
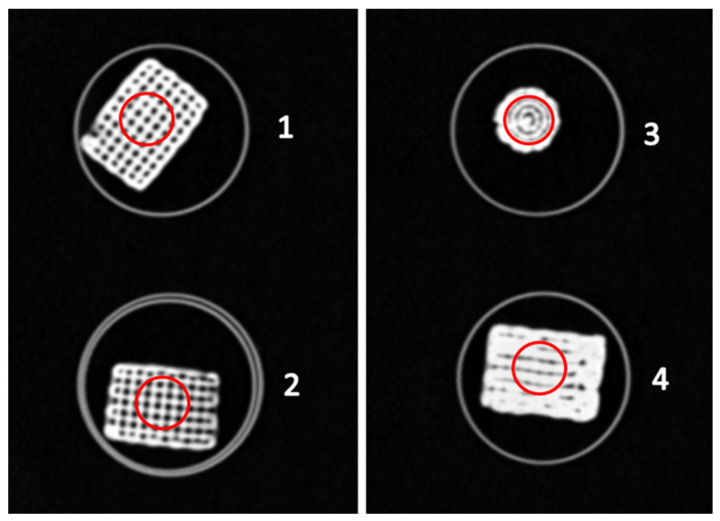
Examples of the CT-scanned scaffolds and selected ROIs for each of the scaffolds. Scaffold #1, Scaffold #2, Scaffold #3, and Scaffold #4.

A slice of the resulting images from the CT scan of the four scaffolds is shown in Figure 5. Furthermore, Figure 5 depicts the placement of the ROIs for the current slice used for measurement of the HUs of the scaffolds.

**Figure 6:**
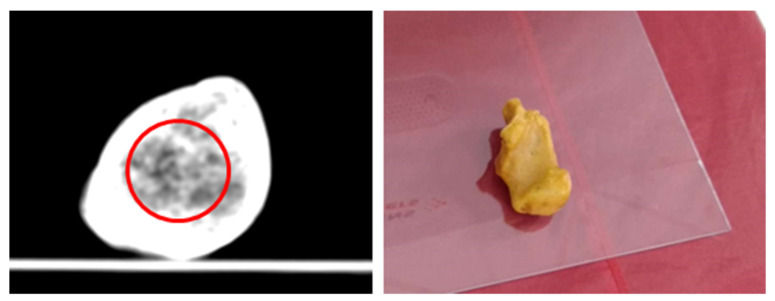
The CT scanned calcaneus bone (**left**) and the calcaneus bone placed on the CT patient bed (**right**).

**Figure 7:**
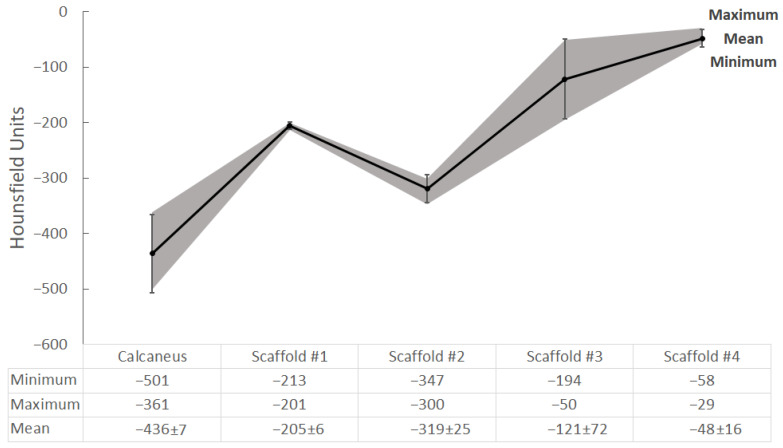
Results from the measurements of the scaffolds and calcaneus bone from the CT scan. The gray field shows the minimum and maximum measured HUs, while the solid black line with the error bars shows the calculated average HUs and the standard deviation, respectively.

A slice of the human calcaneus bone is shown in Figure 6. For measurement of the HUs of the trabecular bone, an area around the facies articularis cuboidea was selected as the ROI. The position of the Ward triangle (an area of a low bone density in the calcaneal bone) was taken into account and avoided as much as possible when selecting the consecutive slices and ROI. The resulting HUs from the measurements performed on the scaffolds and calcaneus bone are shown in Figure 7.

The comparison in Figure 7 shows that Scaffold #2 (mean density = −319 HU) is closest to the calcaneus bone (mean density = −436 HU) compared to the rest of the scaffolds, all of which exhibit higher attenuations. Although there is a noticeable difference in the values, it should be noted that the spongy structure in the scanned calcaneus is less dense than it would be in a live patient. In the current case, the spaces between the trabeculae, normally filled with bone marrow, are empty (occupied by air) after bone cleaning. Having comparable radiodensity to the calcaneus bone provides the opportunity to create a suitable imaging phantom for assessing and improving CT scanning procedures and techniques. On the other hand, a biological scaffold exhibiting densities close to the living tissue would allow for study and comparison of various in vitro scenarios involving bone tissue degeneration and regeneration. In this paradigm, biological testing, histological evaluation, and CT imaging can be used for continuous assessment and optimization of various treatment approaches.

It is worth nothing that the low deviation of the values of Scaffold #1 in the CT scan are due to the much more stable and regular structure of the printed scaffold, visible in Figure 5, compared to the other scaffold samples. As such, it could be used to depict homogeneous tissues.

**Figure 8:**
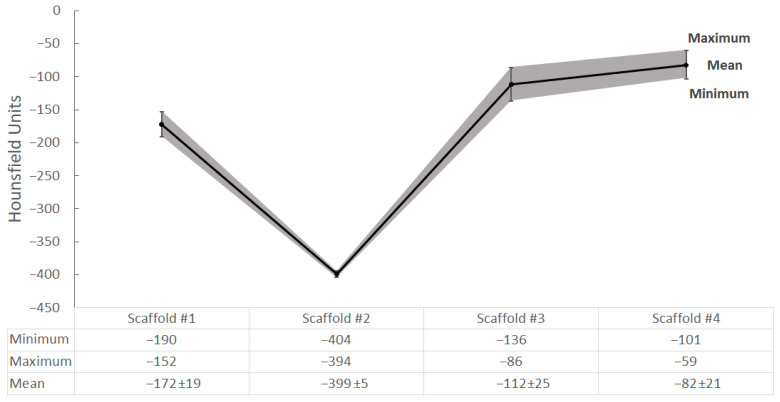
Results from measurements of the scaffolds with the CBCT scan. The gray field shows the minimum and maximum measured HUs, while the solid black line with the error bars shows the calculated average HUs and the standard deviation, respectively.

The results of the CBCT scan can be observed in Figure 8. No comparisons are presented with the bone of the calcaneus for this modality, as it has yet to be scanned in a CBCT system.

The behavior of the examined scaffolds when scanned with CT and CBCT is similar, as can be observed from the shapes of the graphs in Figure 7 and Figure 8. Similar to CT scanning, Scaffold #2 exhibits the lowest density, in this case with a mean of −399 HUs, compared to the several times higher HUs of the other scaffolds. Although there is no information for the CBCT-scanned calcaneus bone, based on observations of the data dynamics (in HUs) in the CT and CBCT images of the scaffolds one could expect the density of the calcaneus bone to be close to that of Scaffold #2.

The differences between the clinical CT and the dental CBCT were expected, and can be explained by the differences in the scanning geometry and reconstruction algorithms, as well as the lower energy and lower slice thickness used for scanning on the CBCT. Further investigations will be performed on the effects and differences between the CT and CBCT modalities when imaging nanosilicate hydrogels for the purpose of complex porous bone-mimicking bioscaffolds.

## 3. Conclusions

In this study, a composite polysaccharide–nanosilicate hydrogel was developed based on Alginate, Methylcellulose, and Laponite, then tested as a bioink for extrusion-based 3D printing.

In the 3D printing tests, the bioink was capable of producing complex porous three-dimensional scaffolds with excellent dimensional accuracy, which was crosslinked into solid objects without further deformation.

The 3D printed scaffolds were cut and mounted as histological slides and stained using standard histological protocols. The polysaccharide chain was visualized as a porous mesh surrounding interconnected spaces with diameters in the range of less than 100 μm and up to 500 μm. The Laponite was visualized as a flaky substance that bound the basic dyes and produced strong background staining. The partial extraction of the hydrogel during tissue processing reduced this background staining. This shows that proper pretreatment may create conditions necessary for further histological and cytological analysis.

The 3D printed scaffolds were scanned with a clinical CT scanner and a dental CBCT scanner, and the resulting images were compared with a CT scan of a dry human calcaneus. The mean density in HUs of the 3D printed scaffolds was close to the natural bone, and could be further tuned by tweaking the Laponite concentration and the porosity of the model. This would make the developed bioink suitable as a tissue-equivalent material for imaging phantoms.

The ability to simulate the biological and radiological properties of the trabecular bone at the same time in both physiological and pathological conditions allows for the production of invaluable multipurpose devices for research into bone pathology and its effects on the imaging properties of engineered bone tissue.

## 4. Materials and Methods

### 4.1. Preparation of the Hydrogel

For the preparation of the hydrogel samples and CaCl_2_ solution, we used the following ingredients:

- 0.3 g. Alginic acid sodium salt from brown algae, middle viscosity, Sigma-Aldrich (Burlington, MA, USA).
- 0.3 g. Methyl Cellulose, viscosity 4000 cP, 2% in H_2_O (20 C) (lit.); Sigma-Aldrich (Burlington, MA, USA).
- 0.3 g Laponite RD; BYK.
- 10 mL deionized water CHROMASOLV™ Plus, for HPLC; Honeywell (Charlotte, NC, USA).
- Calcium chloride, anhydrous, granular; 96%, Sigma-Aldrich (Burlington, MA, USA).

For crosslinking of the hydrogel, we used a solution of CaCl_2_ with a concentration of 100 mmol/L; 1.2 g of CaCl_2_ was diluted in 100 mL deionized water at 36 °C and mixed for 2 min with a magnetic stirrer.

### 4.2. 3D Modelling and 3D Printing

We used a Hyrel 3D Hydra 16A 3D printer (Hyrel 3D, Atlanta, GA, USA) equipped with an SDS10 syringe deposition modular head for extrusion-based 3D printing. We carried out several tests with the three modifications in order to develop the one with optimal qualities for scaffold 3D printing. For the 3D printed tests, we used the following parameters:

- Layer height: 0.5 mm for Tests 1, 2, and 3; 0.4 mm for Tests 4 and 5.
- Shell thickness: 0.8 mm.
- Nozzle diameter: 0.838 mm (for a nozzle, we used commercially available hypodermic needles at 18 G caliber, which were shortened and had the tip ground flat).
- Speed: 10 mm/s for Tests 1 and 2; 5 mm/s for Tests 3, 4, and 5; Perimeters: 4.
- For the generation of g-code, we used Slic3r (Version 1.9) for all tests.
- The 3D models were generated with Autodesk Meshmixer [53] and Autodesk Fusion360 [54], and were exported in stl file format.

### 4.3. Staining

The cured bioscaffolds were cut on a cryostat at 15 μm thickness and −20 °C. The slides were dried at 4 °C. Three staining protocols were applied:

- Diff Quik is a commercial variant of Wright’s stain. In our implementation, we skipped the fixation step with methanol, rehydrated in distilled water for 10 min, and stained sequentially with a buffered solution of Methylene blue and Azure A (nuclear stain) followed by buffered Eosin Y (contrast stain), dehydration, clearing in xylol, and covering.
- Standard Hemalaun–Eosin protocol: rehydration in distilled water for 10 min, staining in Hemalaun for 5 min, fixation and bluing of the He stain in tap water, staining with Eosin, dehydration, clearing in xylol, and covering.
- Staining with Cresyl violet: rehydration in distilled water for 10 min, staining for 1 to 5 min in 0.5% solution of Cresyl violet, differentiation in 1.5% acetic acid in 90% ethanol, dehydration clearing, and covering.

To avoid excessive gel extraction, all slides were stained in the horizontal position.

### 4.4. CT Scanning

For evaluation of the 3D printed scaffolds, we opted for CT scanning with a Siemens SOMATOM Force (Erlangen, Germany). Scanning was performed at 140 kVp, with a slice thickness of 0.5 mm and voxel size of 0.5 mm × 0.5 mm × 0.5 mm × 0.5 mm × 0.5 mm × 0.5 mm.

Moreover, a human calcaneus bone was prepared and scanned with the same CT scanner using the following scanning conditions: energy of 120 kVp, slice thickness of 1 mm, and voxel size of 0.24 mm × 0.24 mm × 1 mm.

The scaffolds were further scanned on a CBCT as well. Scanning was performed on a dental CBCT with an energy of 96 kVp, with a slice thickness of 0.2 mm and voxel size of 0.2 × 0.2 × 0.2 mm. 

For all scanning procedures, the scaffolds were in PMMA containers and placed directly on the patient bed/table. In the case of the calcaneus bone, the sample was placed on a PMMA flat surface on the patient bed.

### 4.5. Radiological Analysis

The datasets were processed with Fiji [56]. Circular regions of interest (ROIs) with similar size were taken for each of the scaffolds, then measurements of three consecutive slices were made for every scaffold. The measurements of each of the consecutive ROIs were averaged and their standard deviations were calculated. Furthermore, the minimum and maximum HUs of the measured regions were accounted for. The same measurements and calculations were made for the calcaneus bone using ROIs with similar size to those for the scaffolds, again taken in three consecutive slices.

## Figures and Tables

**Table 1:** Composition of hydrogel samples M1 and M2.

	Alginate *w*/*v*%	Methylcellulose *w*/*v*%	Laponite *w*/*v*%
Modification 1	3	3	3
Modification 2	5	5	6

**Table 2:** Overall physical characteristics of hydrogel samples M1 and M2.

	*w*/*v*%	Viscosity	Stability	Crosslinking	3D Printing
Modification 1	3/3/3	low	low	low	low
Modification 2	5/5/6	medium	high	excellent	excellent

## Data Availability

The data supporting the reported results are included in the article, and the raw data from the images are available on request.
